# The Relevance of Endothelial Dysfunction Biomarkers in Thalassemia Patients and Healthy Individuals: A Systematic Review and Meta-Analysis

**DOI:** 10.3390/ijms26083842

**Published:** 2025-04-18

**Authors:** Hataichanok Chuljerm, Supawadee Maneekesorn, Gabriel Thorup, Sothida Nantakool, Pimlak Charoenkwan, Kittipan Rerkasem

**Affiliations:** 1 School of Health Sciences Research, Research Institute for Health Sciences, Chiang Mai University, Chiang Mai 50200, Thailand; hataichanok.ch@cmu.ac.th; 2 Division of Hematology and Oncology, Department of Pediatrics, Faculty of Medicine, Chiang Mai University, Chiang Mai 50200, Thailand; supawadee.man@cmu.ac.th (S.M.); pimlak.c@cmu.ac.th (P.C.); 3 Thalassemia and Hematology Center, Faculty of Medicine, Chiang Mai University, Chiang Mai 50200, Thailand; 4 Faculty of Sciences, Brigham Young University—Hawaii, Laie, HI 96762, USA; gabrielleidenthorup@gmail.com; 5 Department of Physical Therapy, Faculty of Associated Medical Sciences, Chiang Mai University, Chiang Mai 50200, Thailand; sothida.n@cmu.ac.th; 6 Environmental–Occupational Health Sciences and Non Communicable Diseases Research Center, Research Institute for Health Sciences, Chiang Mai University, Chiang Mai 50200, Thailand; 7 Clinical Surgical Research Center, Department of Surgery, Faculty of Medicine, Chiang Mai University, Chiang Mai 50200, Thailand

**Keywords:** thalassemia, endothelial dysfunction, cardiovascular diseases, biomarkers

## Abstract

Cardiovascular complications are a major concern in thalassemia patients, primarily driven by endothelial dysfunction. This systematic review and meta-analysis evaluated endothelial biomarkers as indicators of cardiovascular disease risk in thalassemia. A systematic search of PubMed, Scopus, and Embase identified 41 studies comparing biomarkers in thalassemia patients and healthy individuals. The biomarkers analyzed included ICAM-1, VCAM-1, E-selectin, P-selectin, von Willebrand factor (vWF), endothelial microparticles (EMPs), nitric oxide (NO), nitric oxide synthase (NOS), asymmetric dimethylarginine (ADMA), and endothelin-1 (ET-1). Using random effects modeling, pooled standardized mean differences (SMDs) and 95% confidence intervals (CIs) were calculated. The results showed significantly elevated levels of ICAM-1 (SMD 2.15, 95% CI: 1.09–3.22), VCAM-1 (SMD 2.50, 95% CI: 1.35–3.66), E-selectin (SMD 1.21, 95% CI: 0.92–1.50), P-selectin (SMD 1.62, 95% CI: 0.83–2.42), and ET-1 (SMD 1.23, 95% CI: 0.03–2.42) in thalassemia patients. However, NO, ADMA, and vWF showed no significant differences. No studies on NOS were identified, while only one study found significantly elevated EMPs in thalassemia patients. This review highlights ICAM-1, VCAM-1, E-selectin, P-selectin, and ET-1 as key biomarkers for cardiovascular complications in thalassemia. Further research on EMPs and NOS is essential to enhance the understanding of endothelial dysfunction in this population.

## 1. Introduction

Thalassemia is a genetic blood disorder that adversely impacts hemoglobin production in red blood cells, resulting in ineffective erythropoiesis, increased red blood cell hemolysis, and the disrupted iron homeostasis [1,2]. Beta-thalassemia is the predominant form of thalassemia, with a prevalence of 1.5% among the global population [3]. Clinical phenotypes of thalassemia span from nearly normal to severe, necessitating lifelong transfusion support. Despite regular blood transfusions significantly improving the survival of thalassemia patients, the emergence of unnoticed complications persists [4]. Cardiac complications, contributing substantially to mortality in thalassemia patients, primarily stem from an iron overload resulting from blood transfusion, red blood cell hemolysis, and an increased intestinal iron absorption. Excessive iron can be deposited in vital organs, particularly the liver and heart, leading to tissue damage and organ dysfunction. Furthermore, chronic anemia and ineffective erythropoiesis in non-transfusion-dependent thalassemia (NTDT) contribute to a heightened cardiac output and volume overload, subsequently triggering endothelial dysfunction, inflammation, and hypercoagulability [5]. Additionally, oxidative stress induced by iron overload conditions is ultimately implicated in endothelial dysfunction, culminating in vascular remodeling and potential alterations in mechanical properties [4].

Consequently, raising awareness about cardiac complications proves advantageous for thalassemia patients. There are several approaches that are available to assess endothelial dysfunction, a primary contributor to the development of vascular diseases. Among these, the measurement of endothelial biomarkers is a valuable tool for predicting the onset of cardiovascular diseases in thalassemia patients. Various endothelial biomarkers have been used to evaluate the risk of vascular diseases, such as cellular adhesion molecules including intercellular adhesion molecule-1 (ICAM-1), vascular cell adhesion molecule-1 (VCAM-1), E-selectin, and P-selectin. The upregulation of these adhesion molecules promotes leukocyte adhesion to the endothelium, leading to inflammation and endothelial dysfunction, which is one of the mechanisms indicating the development of atherosclerosis [6,7]. Moreover, von Willebrand factor (vWF), a mediator of platelet adhesion to the vascular wall and a key component in thrombus formation, is released into the bloodstream by endothelial cells. Elevated vWF levels can indicate endothelial activation and dysfunction, which consequently poses as a potential risk factor for cardiovascular diseases [8]. Furthermore, the release of endothelial microparticles (EMPs)—small, membrane-bound vesicles derived from the shedding of endothelial cells—serves as a significant biomarker for vascular dysfunction. These microparticles are associated with inflammation processes occurring within the endothelium [9]. In addition, the impaired vasodilation of the endothelium has often been attributed to endothelial dysfunction. Nitric oxide (NO), an essential vasodilatory agent, plays an important role in regulating vascular tone and blood flow. The decrease in NO bioavailability results in endothelial vasodilatory dysfunction. Moreover, the expression of the asymmetric form of dimethylarginine (ADMA), an inhibitor of endothelial nitric oxide synthase (eNOS), subsequently leads to the decrease in NO bioavailability [10]. Furthermore, vascular dysfunction is likely to be the consequence of an imbalance between endothelial-derived constricting and dilating mediators. Thus, the level of endothelin-1 (ET-1), the most potent endogenous vasoconstrictor, can be used to determine endothelial function. The increase in circulating ET-1 leads to the activation of endothelin receptors and contributes to vasoconstriction and vascular remodeling [11,12].

Accordingly, this systematic review and meta-analysis aims to explore the levels of endothelial biomarkers, including ICAM-1, VCAM-1, E-selectin, P-selectin, vWF, EMPs, NO, NOS, ADMA, and ET-1, in both thalassemia patients and healthy individuals.

## 2. Results

### 2.1. Literature Search and Study Characteristics

**Figure 1:**
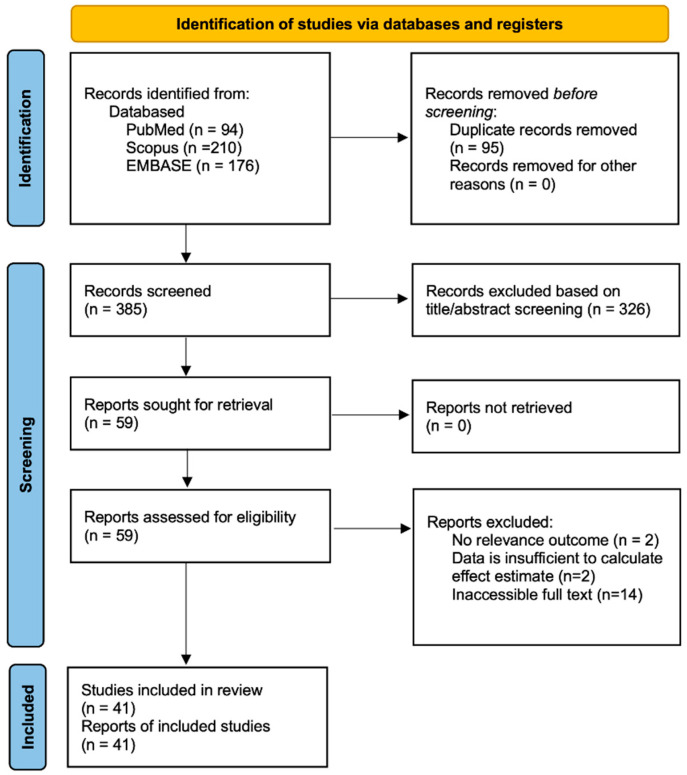
PRISMA flow diagram.

The electronic search, including PubMed, Scopus, and Embase, yielded 480 records. After the removal of 95 duplicate records, 385 records were screened based on the title and abstract. After that, 59 records were assessed for full-text screening. Finally, 41 records met the inclusion criteria, and all records were eligible to be included in the meta-analysis. The PRISMA flow diagram detailing the systematic search process is shown in Figure 1.

### 2.2. Quality Assessment

The quality assessment of all included studies was determined using the Appraisal Tool for Cross-Sectional Studies (AXIS) tool. Out of the 41 included records, *n* = 37 (90.3%) achieved a score of ≥14, which indicated a high quality and low risk of bias. Additionally, *n* = 4 (9.7%) records scored between 60 and 69% (12–13 scores), indicating a moderate quality and risk of bias. And none of the studies had a score below 60%, which signifies a significantly low quality and high risk of bias (Supplementary Table S1).

### 2.3. Study Characteristics

The study characteristics and outcomes of the included records are demonstrated in Table 1. All records included in this review are cross-sectional studies and report at least one of the considered outcomes. The age range of participants across these records varied from a minimum of 4 years to a maximum of 63 years old. Almost all of the included articles studied β-thalassemia patients and β-thalassemia in combination with HbE disease patients. Moreover, some articles investigated both β-thalassemia and Hb H/CS, as well as α-thalassemia and a non-specified type of thalassemia patients. The outcomes of the endothelial biomarkers of interest were reported as mean ± SD values.

### 2.4. Meta-Analysis: Endothelial Biomarkers Level

#### 2.4.1. Intercellular Adhesion Molecule-1 (ICAM-1) Levels

**Figure 2:**
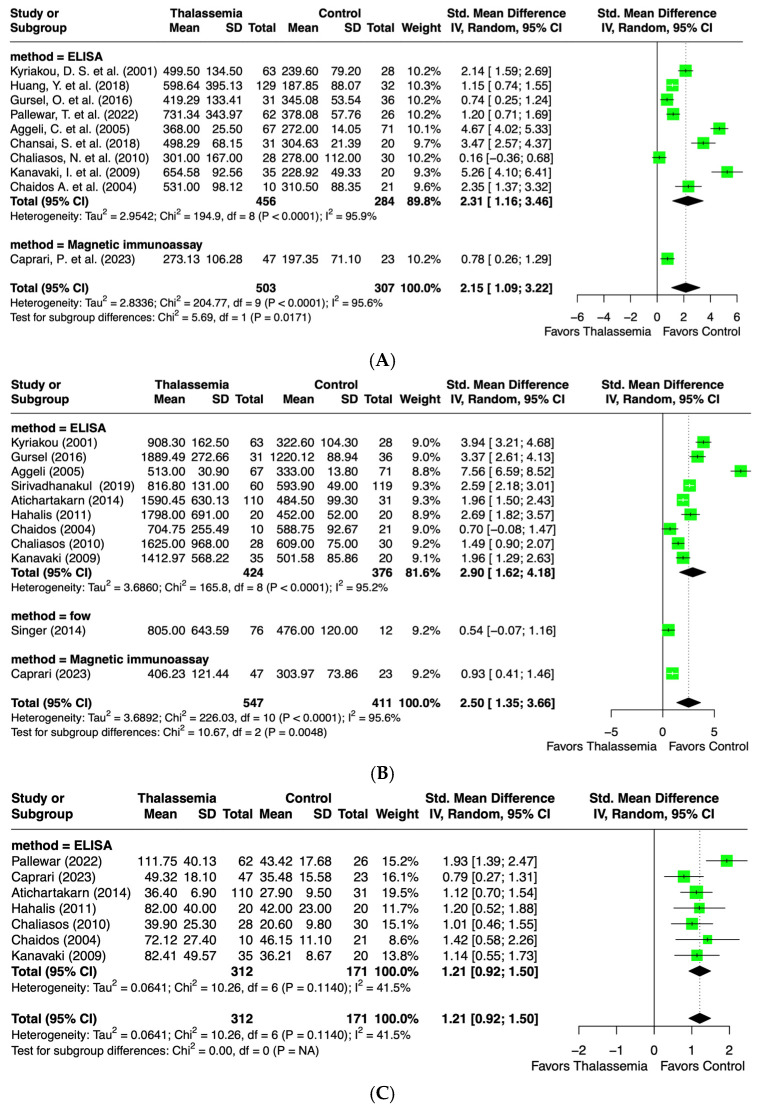

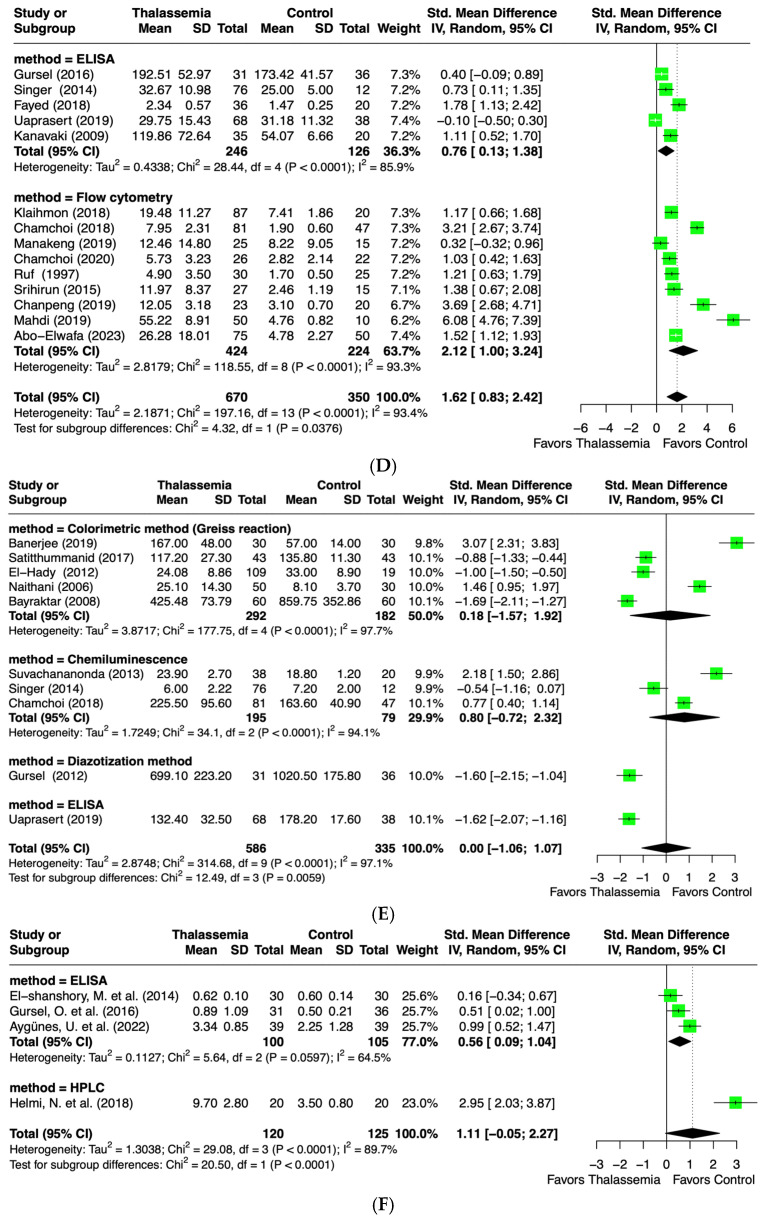

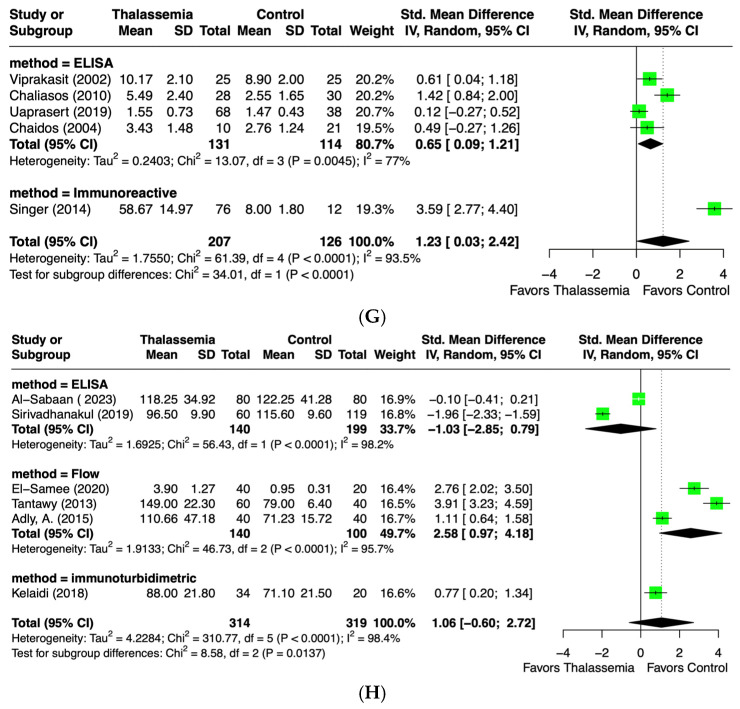
Forest plot showing standard mean difference (SMD) of (**A**) ICAM-1 [17,18,24,26,27,29,45,46,53], (**B**) VCAM-1 [17,24,27,35,36,43,45,46,53], (**C**) E-selectin [26,32,36,43,45,46,53], (**D**) P-selectin [13,21,23,24,25,31,34,39,40,44,46,47,50,51], (**E**) nitric oxide (NO) [14,20,21,22,25,37,38,42,49,50], (**F**) asymmetric dimethylarginine (ADMA) [15,16,24,48], (**G**) endothelin-1 [19,25,45,50,53], and (**H**) von-Willebrand factor (vWF) [28,30,33,35,41,52]. Subgroup analysis is based on methodology. Each study is represented by a green square, the size of which corresponds to the study’s relative weight in the overall analysis. The black diamond illustrates the overall effect size.

A total of 10 records from eligible studies established the levels of ICAM-1 in both thalassemia patients and healthy individuals. All of the studies, 810 participants, were suitable for meta-analysis. Seven of the included studies primarily focused on β-thalassemia and healthy individuals [24,26,27,29,45,46,53], while three studies examined α-thalassemia or unspecified thalassemia types [17,18,32]. ICAM-1 levels were primarily measured using an ELISA (*n* = 9), with one study using a magnetic immunoassay. The ICAM-1 outcome was significantly elevated in the thalassemia group compared to the healthy controls. However, a statistically significant heterogeneity among the studies was noted (I^2^ = 95.6%, *p* < 0.0001). The overall standard mean difference (SMD) was 2.15 (95% CI: 1.09, 3.22). The subgroup analysis based on the methodology (ELISA and magnetic immunoassay) revealed significant heterogeneity in studies using the ELISA method (I^2^ = 95.9%, *p* < 0.0001), with an SMD of 2.31 (95% CI: 1.16, 3.46) (Figure 2A).

#### 2.4.2. Vascular Cell Adhesion Molecule-1 (VCAM-1) Levels

A total of 11 articles investigated the levels of VCAM-1 in both thalassemia patients and healthy individuals. All studies, 958 participants in total, were included for meta-analysis. Six studies focused on β-thalassemia [24,27,43,45,46,53]; three investigated β-thalassemia/Hb E, α-thalassemia, or a combination of β-thalassemia and Hb H/CS [25,35,36]; and two studies examined non-specified thalassemia types [17,32]. The VCAM-1 outcome between the two groups showed significant differences and trended higher in thalassemia compared to the healthy control. However, the heterogeneity between the studies was found to be statistically significant (I^2^ = 95.6%, *p* < 0.0001). The analysis yielded an SMD of 2.50 (95% CI: 1.35, 3.66). The subgroup analysis based on the measurement methods, including the ELISA (*n* = 9), flow cytometry (*n* = 1), and magnetic immunoassay (*n* = 1), showed that significant heterogeneity persisted among studies using the ELISA method (I^2^ = 95.2%, *p* < 0.0001), with an SMD of 2.90 (95% CI: 1.62, 4.17) (Figure 2B).

#### 2.4.3. E-Selectin Levels

A total of seven studies, comprising 485 participants, were included in the meta-analysis of the E-selectin level. Most studies focused on β-thalassemia patients [26,43,45,46,53], with only two studies investigating β-thal/Hb E disease and non-specified types of thalassemia patients [32,36]. Compared with the control, the E-selectin level was significantly higher in the thalassemia group. A moderate, but non-significant heterogeneity among the studies was noted (I^2^ = 41.5%, *p* = 0.11). The analysis revealed an SMD of 1.21 (95% CI: 0.92, 1.50) with all studies using the ELISA method (Figure 2C).

#### 2.4.4. P-Selectin Levels

A total of 14 studies, including 1020 participants, evaluated P-selectin levels and were included in the meta-analysis. Seven studies investigated β-thalassemia/Hb E disease patients [13,21,23,31,39,40,50], six studies were conducted on β-thalassemia [24,34,44,46,47,51], and one study was on β-thalassemia and Hb H/CS patients [25]. The P-selectin level was significantly higher in the thalassemia group compared to the control group. However, a significant heterogeneity was found among studies (I^2^ = 93.4%, *p* < 0.0001). The SMD of the analysis was 1.62 (95% CI: 0.83, 2.42). The subgroup analysis by methodology, including the ELISA (*n* = 5) and flow cytometry (*n* = 9), showed a significant heterogeneity for both the ELISA (I^2^ = 85.9%, *p* < 0.0001) and flow cytometry (I^2^ = 93.3%, *p* < 0.0001) (Figure 2D).

#### 2.4.5. Nitric Oxide (NO) Levels

A total of 10 studies, including 793 participants, were included in the meta-analysis of the NO level. Four studies focused on β-thalassemia/Hb E disease patients [20,21,22,50], four on β-thalassemia [37,38,49,54], one on both β-thalassemia and Hb H/CS patients (28), and one on a non-specific type of thalassemia [14]. The methods used in these studies included the Greiss reaction (*n* = 5), chemiluminescence (*n* = 3), diazotization (*n* = 1), and ELISA (*n* = 1). Interestingly, the pooled data analysis showed no significant difference in NO levels between the thalassemia and healthy control groups. The SMD of the analysis was 0.00 (95% CI: −1.06, 1.07). Nevertheless, in the statistical significance among the studies, heterogeneity was noted (I^2^ = 97.1%, *p* < 0.0001). The methodological subgroup analysis also indicated significant heterogeneity between studies using the Greiss reaction (I^2^ = 97.7%) and chemiluminescence (I^2^ = 94.1%), both with a *p* < 0.0001 (Figure 2E).

#### 2.4.6. Asymmetric Dimethylarginine (ADMA) Levels

A total of four articles investigated the levels of ADMA in both thalassemia patients and healthy individuals. All studies, including 245 participants, were included for meta-analysis. Three included studies primarily focused on β-thalassemia patients [16,24,48] and one on a non-specified type of thalassemia patients [15]. The pooled analysis ADMA outcome trend increased in the thalassemia group, but had no significant difference compared to the healthy control. Significant heterogeneity was observed among studies (I^2^ = 89.7%, *p* < 0.0001), with an SMD of 1.11 (95% CI: −0.05, 2.27). The subgroup analysis based on methodology (ELISA technique, *n* = 3) indicated significant heterogeneity (I^2^ = 64.5%, *p* = 0.0597), with an SMD of 0.56 (95% CI: 0.09, 1.04) (Figure 2F).

#### 2.4.7. Endothelin-1 (ET-1) Levels

A total of five studies, including 333 participants, were included in the meta-analysis of the endothelin-1 level. Two articles focused on β-thalassemia patients [45,53], two on β-thalassemia/Hb E disease [19,50] and one on both β-thalassemia and Hb H/CS patients [25]. Compared with the control, the endothelin-1 levels were significantly higher in the thalassemia group. However, the statistically significant heterogeneity among the studies was remarkable (I^2^ = 93.5%, *p* < 0.0001), with an SMD of 1.23 (95% CI: 0.03, 2.42). The methodological subgroup analysis (ELISA, *n* = 4) also revealed significant heterogeneity (I^2^ = 77%, *p* = 0.045) and an SMD of 0.65 (95% CI: 0.09, 1.21) (Figure 2G).

#### 2.4.8. Von Willebrand Factor (vWF) Levels

A total of six articles investigated the levels of vWF in thalassemia patients and healthy individuals. All studies, 633 participants in total, were included for meta-analysis. Most studies investigated β-thalassemia patients [28,33,41,52,55], with only one on α-thalassemia patients [35]. The methods used included the ELISA (*n* = 2), flow cytometry (*n* = 3), and immunoturbidimetry (*n* = 1). The pooled analysis of the vWF outcome showed no significant increase in thalassemia compared to the control group. A significant heterogeneity was observed among studies (I^2^ = 98.4%, *p* < 0.0001), with an overall SMD of 1.06 (95% CI: −0.60, 2.72). The methodological subgroup analysis revealed significant heterogeneity for the ELISA (I^2^ = 98.2%, *p* < 0.0001, SMD = −1.03; 95% CI: −2.85, 0.79) and flow cytometry (I^2^ = 95.7%, *p* < 0.0001, SMD = 2.58; 95% CI: 0.97, 4.18) (Figure 2H).

#### 2.4.9. Endothelial Microparticles (EMPs)

A total of one study investigated the levels of EMPs in thalassemia patients and in a healthy control [52]. The meta-analysis was not able to be performed with this parameter. In this study, 40 beta-thalassemia patients and 40 healthy individuals were recruited. The average ages were 15.4 ± 3.9 and 14.7 ± 2.1 years in the thalassemia group and healthy control, respectively. The circulating EMPs were measured using flow cytometry. The findings revealed that the expression of EMPs in thalassemia patients was 1.97 ± 1.21%, which is significantly higher than those of the control (1.05 ± 0.46%).

### 2.5. Publication Bias of Included Studies

**Figure 3:**
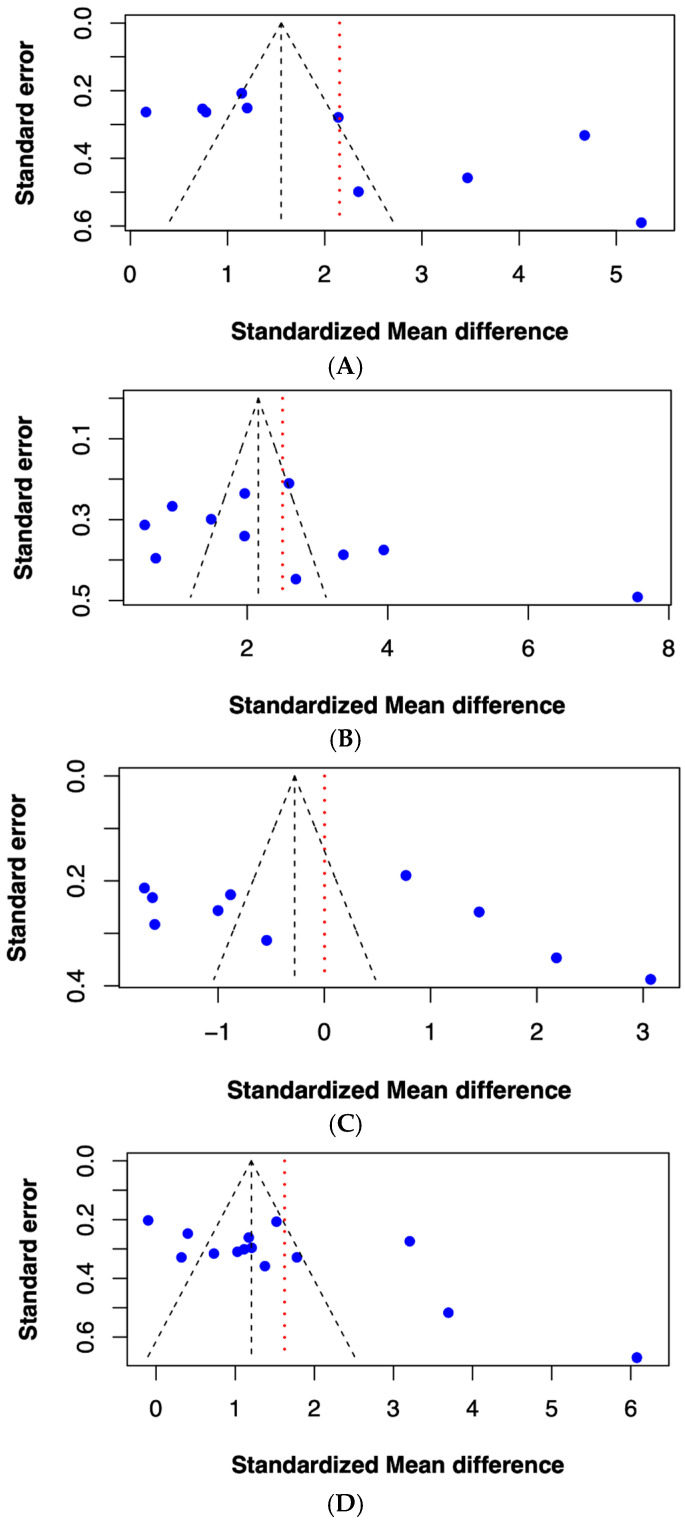
Funnel plot for analysis of (**A**) ICAM-1, (**B**) VCAM-1, (**C**) nitric oxide, and (**D**) P-selectin. The black dashed vertical line indicates the pooled effect estimate. The black dashed triangle outlines the expected 95% confidence region under the assumption of no publication bias. The red dashed vertical line represents the adjusted effect size after accounting for potential publication bias. The blue dots represent individual studies included in the meta-analysis.

Publication bias was assessed using a linear regression test of funnel plot asymmetry and was interpreted by Egger’s test. The funnel plot method is recommended for evaluating publication bias when a meta-analysis includes at least 10 effect sizes, as fewer studies reduce the statistical power [56,57]. Therefore, the studies that reported on ICAM-1, VCAM-1, NO, and P-selectin were selected for this analysis. The results demonstrated a significant funnel plot asymmetry in studies on ICAM-1 and P-selectin (*p* < 0.05) (Figure 3A,D), indicating a potential publication bias. In contrast, the analyses for VCAM-1 and NO showed no significant asymmetry (*p* > 0.05) (Figure 3B,C), suggesting an absence of publication bias for these biomarkers.

## 3. Discussion

The major finding in this systematic review and meta-analysis is that the blood biomarkers indicating endothelial dysfunction are associated with thalassemia, as opposed to healthy individuals. In thalassemia patients, iron overload is the primary cause of toxicity, affecting several vital organs, including the cardiovascular system. The excess iron promotes the production of reactive oxygen species (ROS) through the Fenton reaction, leading to inflammation and tissue damage [58,59]. The oxidative stress and inflammatory condition in thalassemia patients can stimulate the expression of endothelial adhesion molecules, such as ICAM-1, VCAM-1, E-selectin, and P-selectin, which promote the recruitment of circulating leukocytes to the vessel wall and exacerbate endothelial inflammation [60]. The overexpression of these molecules is related to endothelial activation and dysfunction, which is the initial stage of cardiovascular diseases like atherosclerosis [61]. Furthermore, there is evidence suggesting that an increased ROS production can promote endothelial dysfunction [7]. This meta-analysis found that the expression levels of endothelial adhesion molecules, including ICAM-1, VCAM-1, E-selectin, and P-selectin, were significantly elevated in thalassemia patients compared to healthy individuals. Notably, serum ferritin—a widely used surrogate marker of total body iron stores—was found in several of the included studies to be significantly higher in thalassemia patients and to correlate positively with these adhesion molecules, suggesting that iron overload may play a causative role in endothelial damage [17]. These findings underscore the role of iron-induced oxidative stress and inflammation in triggering endothelial activation. Thus, inflammatory activation and endothelial dysfunction are significant contributors to the development and progression of cardiovascular diseases, which are the leading cause of death among thalassemia patients. Despite this, the true incidence of atherosclerosis in this population is challenging to determine due to several confounding factors, including survivor bias, differences in iron chelation therapy, and the limited scale and scope of available clinical studies. Historically, atherosclerotic cardiovascular disease (ASCVD) has been considered rare among individuals with thalassemia, largely because of the reduced life expectancy and other competing mortality risks. However, this perspective is evolving. Advances in therapeutic strategies—particularly the widespread adoption of iron chelation therapy—have significantly extended the life expectancy of patients with both β-thalassemia major (TM) and intermedia (TI), thereby increasing the relevance of long-term complications, such as ASCVD [62,63]. Given that endothelial dysfunction often precedes the clinical onset of cardiovascular disease, the routine monitoring of endothelial biomarkers in thalassemia patients could provide a valuable insight into early vascular alterations, facilitating timely intervention and potentially improving long-term cardiovascular outcomes. Previous studies have suggested that measuring the levels of ICAM-1, VCAM-1, E-selectin, and P-selectin can effectively reflect the inflammatory processes occurring within the endothelium [61,64]. Therefore, the upregulation of these endothelial adhesion molecules in thalassemia patients should be closely monitored—not only to assess the current vascular status but also to guide proactive strategies aimed at preventing complications, particularly cardiovascular diseases that may emerge as patients age.

In addition, vWF is often regarded as a marker of endothelial damage; however, its role remains controversial. This systematic review and meta-analysis found no significant differences in the pooled analysis of vWF levels between thalassemia patients and healthy individuals. Several factors may account for the variability in the vWF expression observed among thalassemia patients. For instance, serum ferritin levels have been shown to correlate with vWF levels, with patients with serum ferritin concentrations >2500 µg/mL exhibiting significantly higher vWF levels compared to those with serum ferritin levels <2500 µg/mL [52]. Additionally, other variables, such as the splenectomy status and patient age, may influence vWF levels in this population [65,66]. This finding suggests that vWF may not serve as a reliable biomarker for endothelial damage and dysfunction in thalassemia patients.

Endothelial dysfunction can be characterized by a decrease in nitric oxide (NO) production, disrupting the balance between vascular relaxation and contraction [67]. Under normal conditions, NO is produced from L-arginine in endothelial cells by the enzyme endothelial nitric oxide synthase (eNOS). It is released from the endothelium to maintain vascular homeostasis, primarily through its vasodilatory effects [68,69]. Additionally, NO can inhibit the expression of adhesion molecules on the endothelial surface, reducing leukocyte adhesion and inflammation. Consequently, a reduction in NO levels contributes to endothelial dysfunction and increases the risk of cardiovascular diseases [70]. However, this systematic review and meta-analysis found no significant decrease in NO levels between thalassemia patients and healthy controls. While NO levels in thalassemia patients are generally reported to be lower, some studies have observed increased levels of NO compared to healthy individuals. Several factors may contribute to these conflicting findings of NO levels in thalassemia patients, including disease severity, hemolysis, iron overload, and oxidative stress. In the case of severe hemolysis, cell-free hemoglobin in the blood circulation can scavenge NO, reducing its bioavailability. In addition, lysed red blood cells can release arginase, an enzyme that degrades arginine. The depletion of arginine further decreases the bioavailability of NO, impairing its protective effect and contributing to endothelial dysfunction [71]. However, increased NO levels have also been observed in some studies involving thalassemia patients who receive multiple blood transfusions and are treated with iron chelators. Previous research suggests that the improved oxygen transport capacity following blood transfusions may enhance NO production. Hypoxia, a condition common in thalassemia, can suppress endothelial nitric oxide synthase (eNOS) activity. Thus, restoring normal blood oxygenation through regular transfusions may stimulate NO synthesis [72]. Additionally, evidence indicates that standard iron chelators, such as deferoxamine (DFO), can enhance eNOS activity, potentially contributing to increases in NO production [73]. Despite these findings, this systematic review and meta-analysis identified a significant gap in the studies directly investigating eNOS levels in biological samples from thalassemia patients compared to healthy individuals.

Additionally, oxidative stress triggered by iron overload in thalassemia patients also enhances the production of asymmetric dimethylarginine (ADMA), an endogenous competitive inhibitor of eNOS. ADMA competes with L-arginine for the same binding site on eNOS, resulting in a decreased NO production [67]. Under conditions of restricted L-arginine availability, nitric oxide synthase may undergo a shift towards generating superoxide radicals instead of NO, contributing to endothelial inflammation and damage. [74]. Previous evidence has shown that hemolysis-associated conditions, including thalassemia, typically exhibit elevated levels of ADMA, due to the releases of stored ADMA from red blood cells during hemolysis [75]. Additionally, elevated ADMA levels can be observed in conditions such as hypercholesterolemia, atherosclerosis, hypertension, chronic kidney diseases, and diabetes mellitus, making ADMA a potential risk predictor for cardiovascular disease [76]. This systematic review and meta-analysis found a trend toward increased ADMA levels in thalassemia patients compared to healthy individuals. However, a key limitation of this study is the lack of evidence examining both NO and ADMA levels within the same cohort of thalassemia patients. Such studies are crucial for gaining insight into the pathogenesis of cardiovascular complications in thalassemia patients.

Under physiological conditions, the balance between NO, a vasodilator, and endothelin-1 (ET-1), a potent endogenous vasoconstrictor, plays a crucial role in maintaining vascular tone and blood pressure. An imbalance between vasodilators and vasoconstrictors is indicative of endothelial dysfunction and presents as an early event in the development of cardiovascular diseases [10]. This systematic review and meta-analysis revealed that elevated levels of ET-1 in thalassemia patients are associated with a reduced NO bioavailability. A previous study has demonstrated that ET-1 can disrupt the expression and activity of NO synthase, resulting in a decreased NO production [77]. Furthermore, hypoxia has been identified as a key inducer of ET-1 production. Chronic anemia in thalassemia patients, driven by ineffective erythropoiesis and hemolysis, leads to tissue hypoxia. In response, endothelial cells are stimulated to release large amounts of hypoxia inducible factor (HIF) 1α which subsequently upregulates the expression of its target gene, including ET-1 [78,79]. Notably, levels of ET-1 have been reported to be higher in non-transfusion-dependent thalassemia patients compared to transfusion-dependent patients, likely due to the different degrees of hypoxia and anemia [80]. In addition to the regulation of vascular tone, ET-1 also stimulates the release of inflammatory cytokines, including tumor necrosis factor-alpha (TNF-α) and interleukin-6 (IL-6), promotes free radical formation, and facilitates platelet activation [81]. These processes contribute to vascular inflammation, oxidative stress, and thrombosis. In summary, elevated ET-1 levels in thalassemia patients are indicative of endothelial dysfunction and may increase the risk of cardiovascular complications. The monitoring and management of ET-1 levels are important for thalassemia patients.

Additionally, endothelial microparticles (EMPs) are considered a surrogate marker for endothelial dysfunction and vascular remodeling, as they are shed from the plasma membrane of activated endothelial cells [82,83]. Alterations in circulating EMP levels may reflect pathological mechanisms in various diseases, including cardiovascular diseases. Despite their significance, few studies have investigated EMP levels in thalassemia patients. This systematic review and meta-analysis identified only one study, conducted by Adly et al., that compared EMP levels in thalassemia patients to healthy individuals [52]. This gap in the literature highlights the importance of further investigating EMP levels in thalassemia, considering their potential as a specific biomarker for endothelial damage [84]. Additionally, EMP levels are associated with inflammation status. Investigating the EMP levels may provide valuable information about the relationship between endothelial dysfunction, inflammation, and disease progression in thalassemia patients.

This systematic review and meta-analysis comprehensively evaluated key endothelial biomarkers as potential indicators of cardiovascular complications in thalassemia patients. It identifies suitable endothelial biomarkers that can be used as indicators for cardiovascular diseases in this population. However, certain limitations warrant consideration. The predominance of cross-sectional study designs limits the ability to assess biomarker levels over time and their relationship with disease progression. Additionally, variability in the types of thalassemia patients included and the influence of treatment regimens contribute to significant heterogeneity, limiting the generalizability of the findings. Another potential source of heterogeneity arises from the variation in the iron chelation strategies employed across the included studies. The use of different chelators—such as deferoxamine (DFO), deferiprone (DFP), deferasirox (DFX), or their combinations—can influence the iron burden to varying degrees, thereby potentially affecting the expression levels of endothelial biomarkers. However, due to the inconsistent or incomplete reporting of iron chelation regimens, this variable could not be systematically analyzed, limiting the ability to assess its impact on the observed outcomes. Moreover, the limit number of studies investigating certain biomarkers precluded the use of a funnel plot asymmetry test, leading to the lack of a formal publication bias analysis. This limitation may lead to an overestimation of the effect sizes and affect the reliability of the results. Addressing these methodological challenges in future research is essential to strengthen the validity and applicability of endothelial biomarkers for assessing cardiovascular risks in thalassemia patients.

## 4. Materials and Methods

### 4.1. Information Source and Search Strategy

This systematic review and meta-analysis was conducted in accordance with the Preferred Reporting Items for Systematic Review and Meta-Analysis (PRISMA) guidelines (see Supplementary Table S2) [85]. This study protocol was registered in the PROSPERO database (CRD42023431232). An amendment was made to the protocol to include additional endothelial biomarkers as study outcomes. The literature search was conducted by two independent reviewers. Three electronic databases, including PubMed, Scopus, and EMBASE, were systematically searched on 4 September 2024. Search terms were as follows: (thalassemia) AND (‘intercellular adhesion molecule-1’ OR ‘ICAM1’ OR ‘ICAM-1’ OR ‘vascular cell adhesion molecule-1’ OR ‘VCAM1’ OR ‘VCAM-1’ OR ‘sVCAM1’ OR ‘soluble VCAM1’ OR ‘Endothelial leucocyte adhesion molecule-1’ OR ‘ELAM-1’ OR ‘ELAM1’ OR ‘E-selectin OR ‘CD62 antigen-like family member E’ OR ‘CD62E’ OR ‘leukocyte-endothelial cell adhesion molecule-2’ OR ‘nitric oxide’ OR ‘NO’ OR ‘nitric oxide synthase’ OR ‘NOS’ OR ‘asymmetric form of dimethylarginine’ OR ‘ADMA’ OR ‘endothelial microparticles’ OR ‘EMPs’ OR ‘endothelial microparticle’ OR ‘EMP’ OR ‘Von willebrand factor’ OR ‘vWF’ OR ‘P-selectin’ OR ‘CD62P’ OR ‘Granule Membrane Protein 140’ OR ‘GMP-140’ OR ‘Platelet Activation-Dependent Granule to External Membrane Protein’ OR ‘PADGEM’ OR ‘endothelin-1’ or ‘ET-1’ or ‘preproendothelin-1’ or ‘PPET1’). All of which were linked with the Boolean operators ‘AND’ and ‘OR’.

### 4.2. Study Selection

After completing the search process, duplicate records were removed. Two independent reviewers (HC and SM) screened the results of the initial search by title and abstract. Then, the full text of the relevant study was investigated based on inclusion and exclusion criteria. The inclusion criteria of this study were as follows: (1) cross-sectional study related to endothelial biomarkers in thalassemia and (2) detected biomarkers in fluids (e.g., serum and plasma). The exclusion criteria were as follows: (1) multiple duplicate data in different works, (2) reviews, letters, editorials, expert opinions, talks, and systematic reviews, and (3) studies that included thalassemia patients with coexisting diseases. The eligible studies were required to be accessible full-text articles. The disagreements of eligible studies were resolved through discussion with the third reviewer (KR).

### 4.3. Data Extraction

The following data from eligible studies were extracted by three independent reviewers (HC, SM, and GT): (1) publication details (name of the first author, year of publication, study design, and location), (2) characteristics of participants (number of participants and mean age), and (3) outcome of endothelial biomarkers level (ICAM-1, VCAM-1, E-selectin, P-selectin, nitric oxide (NO), nitric oxide synthase (NOS), ADMA, EMP, endothelin-1, and vWF). Disagreements were resolved by consultation with a third reviewer if needed (KR).

### 4.4. Quality Assessment

The quality of eligible studies was checked by two independent authors (HC and SM) using the Appraisal Tool for Cross-Sectional Studies (AXIS) [86]. The 20 AXIS questions include 5 main quality criteria to appraise objectives, methods, results, discussion, ethics, and funding of eligible studies. Each item of the 20 AXIS questions was scored by assigning a numeric value to two categories of response: YES (score as 1) and NO (score as 0). The quality of the publication was assessed according to the total appraisal scores: high quality (equal to or exceeding 70% of the total score, or a score ≥ 14), moderate quality (publication score between 60 and 69%), and low quality (publication score below 60%) [87]. If there was no consensus on an item, the third reviewer (KR) was responsible for making a final decision.

### 4.5. Statistical Analysis and Publication Bias

A minimum of two included studies were considered to pool in the meta-analysis. The random effects model was used as an approach to calculate the pooled standardized mean difference (SMD) with 95% confidence intervals (CIs) between thalassemia patients and healthy controls. For studies that reported the values of biomarkers as median and range, the mean and standard deviation (SD) were estimated by the formula developed by Wan et al. [88]. The heterogeneity of included evidence was calculated using the I^2^ statistic (I^2^ > 60% implies statistically significant heterogeneity). R program version 4.4.1, metafor package were used for the meta-analysis. The publication bias of each endothelial biomarker was determined using a funnel plot and interpreted with Egger’s test.

## 5. Conclusions

**Figure 4:**
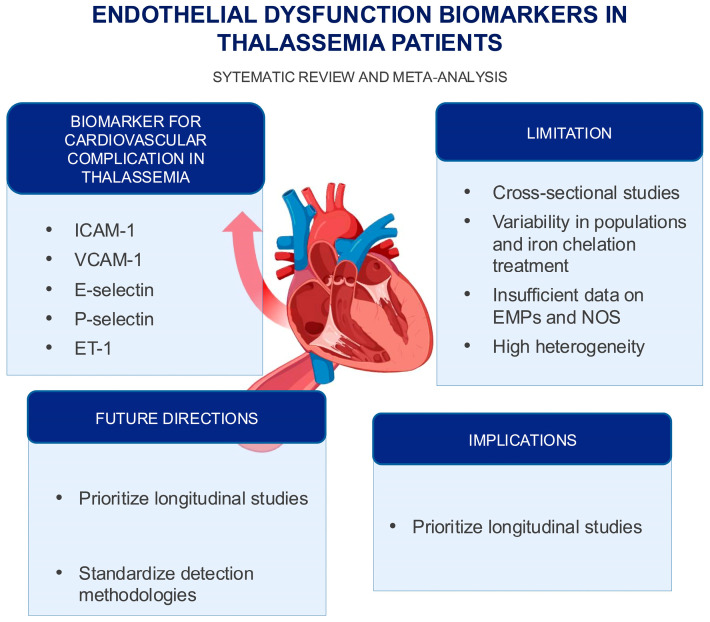
Summary of systematic review and meta-analysis on endothelial biomarkers in thalassemia patients.

This systematic review and meta-analysis identifies ICAM-1, VCAM-1, E-selectin, P-selectin, and ET-1 as biomarkers for assessing cardiovascular risk in thalassemia patients, underscoring their potential for early detection and targeted interventions in clinical practice. However, the findings are limited by the predominance of cross-sectional study designs, the variability in patient populations and treatments, and the insufficient data on certain biomarkers, like EMPs and NOS. The high heterogeneity across studies and the absence of a formal publication bias assessment may affect the reliability of the results. Future research should prioritize longitudinal studies and standardize detection methodologies to improve the generalizability of the findings (Figure 4).

## Figures and Tables

**Table 1:** Study characteristics and the outcome of included articles.

	Study ID	Study Group		Group Size (N)	Age (Years)	Serum Ferritin (ng/mL)	ICAM-1 (ng/mL)	VCAM-1 (ng/mL)	Nitric Oxide (µM)	ADMA (µmol/L)	EMP (%)	vWF	P-Selectin	E-Selectin (ng/mL)	Endothelin-1 (pg/mL)
1	Klaihmon [13]	β-thal/HbE		87	12.8 ± 4.9	BMT = 1795.1							19.48 ± 11.27%		
		Healthy control		20	12.4 ± 3.9	ND							7.41 ± 1.86%		
2	Banerjee [14]	Thalassemia		30	ND	200–870			167.0 ± 48.0						
		Healthy control		30	ND	19.04–143.84			57.0 ± 14.0						
3	Helmi [15]	Thalassemia		20	ND	ND				0.048 ± 0.014					
		Healthy control		20	ND	ND				0.017 ± 0.004					
4	El-shanshory [16]	β-TM		30	6–18	600–5000				0.62 ± 0.10					
		Healthy control		30		100–150				0.60 ± 0.14					
5	Kyriakou [17]	Thalassemia		63	24.3 (10–56)	TM = 4186 TI = 356 β-thalassemia carriers = 85	499.5 ± 134.5	0.91 ± 0.16							
		Healthy control		18	28 (10–60)	ND	239.6 ± 79.2	0.32 ± 0.10							
6	Huang [18]	Thalassemia	β-TM	67	9.5 ± 4.6	3814 (380–15,960)	808.1 ± 614.6								
			β-TI	22	14.5 ± 7.8	2552 (126–12,169)	629.6 ± 334.8								
			α-TI	33	18.2 ± 14.2	544 (51–11,110)	613.1 ± 351.9								
			α + β	7	8.5 ± 2.9	2915 (358–6446)	343.9 ± 118.9								
		Healthy control		32	17.5 ± 13.3	ND	187.8 ± 88.1								
7	Viprakasit [19]	β-thalassemia/HbE		25	5–15	ND									10.17± 2.1
		Healthy control		25	5–15	ND									8.9 ± 2.0
8	Suvachananonda [20]	β-thalassemia/HbE		38	12.0 ± 1.9	Mild = 708.8 ± 276.4 Moderate = 1953.0 ± 307.3 Severe = 2162.0 ± 360.4			23.9 ± 2.7						
		Healthy control		20	11.2 ± 0.1	ND			18.8 ± 1.2						
9	Chamchoi [21]	β-thal/HbE	NSP	58	ND	633.1 (336.8 ± 1552.0)			0.23 ± 0.95				4.8 ± 1.5%		
			SP	23	ND	1428.0 (606.5 ± 2575.0)			0.22 ± 0.95				11.1 ± 2.9%		
		Healthy control		47	ND	ND			0.164 ± 0.41				1.9 ± 0.6%		
10	Satitthummanid [22]	β-thal/HbE		43	34.63 ± 9.7	3090.2 ± 3297			117.2 ± 27.3						
		Healthy control		43	36.69 ± 8.6	ND			135.8 ± 11.3						
11	Manakeng [23]	β-thal/HbE	PAH	11	39 ± 11	769 ± 791							13.45 ± 16.88%		
			No PAH	14	37 ± 12	1072 ± 981							11.69 ± 13.54%		
		Healthy control		15	28 ± 5	62 ± 50							8.22 ± 9.05%		
12	Gursel [24]	β-TM		31	8.5 ± 0.1	2235 (390–5119)	419.29 ± 133.41	1889.49 ± 272.66		0.89 ± 1.09			192.51 ± 52.97 ng/mL		
		Healthy control		36	8.0 ± 0.3	32 (18–90)	345.08 ± 53.54	1220.12 ± 88.94		0.50 ± 0.21			173.42 ± 41.57 ng/mL		
13	Singer [25]	Thalassemia	TM	41	27.5 ± 9.8	TRV < 2.5 m/s = 1872 (2534) TRV > 2.5 m/s = 486 (355)		1043 ± 840	9.1 ± 12				35 ± 13 ng/mL		38 ± 29
			TI	27	30.5 ± 12.7			692 ± 600	4.9 ± 2.5				30 ±12 ng/mL		65 ± 45
			Hb-Cs	8	23.2 ± 3.7			680 ± 422	4.0 ±1.9				33 ± 7 ng/mL		73 ± 49
		Healthy control		12	20 ± 35	ND		476 ±120	7.2 ± 2				25 ± 5 ng/mL		8 ±1.8
14	Pallewar [26]	β-thalassemia major		62	10.7 ± 3.1	3168.70 ± 1361.30	731.34 ± 343.97							111.75 ± 40.13	
		Healthy control		26	9.6 ± 3.5	ND	378.08 ± 57.76							43.42 ± 17.68	
15	Aggeli [27]	β-TM		67	24.6 ± 0.7	1108 ± 38.17 pmol/L	368 ± 25.5	513 ± 30.9							
		Healthy control		71	25.63 ± 0.49	312 ± 15.09 pmol/L	272 ±14.1	333 ± 13.8							
16	Al-Sabaan [28]	β-TM		80	ND	ND						118.25 ± 34.92 U/dL			
		Healthy control		80	ND	ND						122.25 ± 41.28 U/dL			
17	Chansai [29]	β-thal	NSP	20	14.9 ± 1.0	876 (734.91–1229.39)	520.82 ± 55.22								
			SP	11	16.5 ± 1.7	1133 (880–1473.82)	475.75 ± 79.0								
		Healthy control		20	21.4 ± 0.5	ND	304.63 ± 21.39								
18	Tantawy [30]	β-TM		60	10.5 ± 3.9	2774.5 ± 988.9						149 ± 22.3%			
		Healthy control		40	9.7 ± 3.1	176 ± 53						79 ± 6.4%			
19	Chamchoi [31]	β-thal/HbE	NSP	16	34 (24–40)	629 (339–1458)							5.33 ± 3.00%		
			SP	10	24 (18–28)	1298 (216–2625)							6.13 ± 3.70%		
		Healthy control		22	27 (25–31)	45 (28–97)							2.82 ± 2.14%		
20	Caprari [32]	Thalassemia		47	39 ± 15	662 (67–4438)	273.13 ± 106.28	406.23 ± 121.44						49.32 ± 18.1	
		Healthy control		23	40 ± 11	ND	197.35 ± 71.10	303.97 ± 73.86						35.48 ± 15.58	
21	El-Samee [33]	β-thalassemia		40	23 ± 4.0	ND						3.90 ± 1.27 IU/dL			
		Healthy control		20	23.3 ± 4.6	ND						0.95 ± 0.31 IU/dL			
22	Ruf [34]	β-TM		30	24 (12–42)	ND							4.9 ± 3.5%		
		Healthy control		25	24 (15–40)	ND							1.7 ± 0.5%		
23	Sirivadhanakul [35]	α-thal	Non-deletion	31	12.9 ± 4.8	209.2 (103.0–353.6)		816.8 ± 131.0				96.5 ± 9.9%			
			Deletion	29	13.3 ± 4.4	64.5 (41.1–103.1)									
		Healthy control		119	13.6 ± 3.0	56.6 (41.1–77.0)		593.9 ± 49.0				115.6 ± 9.6%			
24	Atichartakarn [36]	β-thal/HbE	SP	61	29.2 ± 10.5	1892.1 (97.9–9520)		1575.9 ± 557.4					113.4 ± 31.2 ng/mL	43.3 ± 21.5	
			NSP	49	33.9 ± 11.4	1050 (17.2–7813)		1605 ± 696.3					56.4 ± 32.1 ng/mL	29.5 ± 18.4	
		Healthy control		31	ND	10–300		484.5 ± 99.3					ND	27.9 ± 9.5	
25	El-Hady [37]	β-thalassemia		109	7.25 ± 2.42	1788.32 ± 362.59			24.08 ± 8.86						
		Healthy control		19	7.05 ± 2.48	37.4 ± 20			33 ± 8.9						
26	Naithani [38]	TD-β-thal		50	10.2 ± 4.6	3709 ± 1625			25.1 ± 14.3						
		Healthy control		30	9.0 ± 4.91	131 ± 117			8.1 ± 3.7						
27	Srihirun [39]	β-thal/HbE		27	32.2 ± 5.42	TRV < 2.5 m/s = 1414 TRV > 2.5 m/s = 587.7							11.97 ± 8.37%		
		Healthy control		15	25.25 ± 0.9	ND							2.46 ± 1.19%		
28	Chanpeng [40]	β-thal/HbE	NSP	15	28.7 ± 6.2	512.2 ± 319.6							17.7 ± 4.2%		
			SP	8	34.0 ± 8.8	1757 ± 382.8							6.4 ± 1.6%		
		Healthy control		20	28.0 ± 6.1	76.2 ± 37.8							3.1 ± 0.7%		
29	Kelaidi [41]	β-thalassemia		34	37.6 ± 12.9	789.9 ± 514.5						88.0 ± 21.8 IU/dL			
		Healthy control		20	Age matched	ND						71.1 ± 21.5 IU/dL			
30	Gursel [42]	β-TM		31	10.84 ± 4.38	2235 (390–5119)			699.1± 223.2						
		Healthy control		36	10.06 ± 4.38	32 (18–90)			1020.5 ± 175.8						
31	Hahalis [43]	β-TI		20	35.9 ± 11.6	1568 ± 1344		1798 ± 691						82 ± 40	
		Healthy control		20	34.5 ± 5.4	ND		452 ± 52						42 ± 23	
32	Mahdi [44]	β-thal	minor	10	ND	ND							5.25 ± 3.912%		
			intermedia	10	ND	ND							87.20 ± 8.091%		
			major	30	ND	ND							73.20 ± 12.548%		
		Healthy control		10	ND	ND							4.76 ± 0.823%		
33	Chaliasos [45]	β-TM		28	25.2 ± 13.0	1286 ± 839	301 ± 167	1625 ± 968						39.9 ± 25.3	5.49 ± 2.40
		Healthy control		30	Age matched	ND	278 ± 112	609 ± 75						20.6 ± 9.8	2.55 ± 1.65
34	Kanavaki [46]	β-TI		35	34.7 ± 15.9	650.6 ± 523.7	654,580 ± 9256	1412.97 ± 568.22 (mg/L)					119.86 ± 72.64 mg/L	82,410 ± 49,570	
		Healthy control		20	Age matched	ND	228,920 ± 49,330	501.58 ± 85.86 (mg/L)					54.07 ± 6.66 mg/L	36,210 ± 8670	
35	Fayed [47]	β-thalassemia		36	9.9 ± 4.7	1623 ± 1038							2.337 ± 0.57 ng/mL		
		Healthy control		20		90.4 ± 23.2							1.467 ± 0.25 ng/mL		
36	Aygüneş [48]	β-thalassemia major		39	6.85 ± 0.49	1394.7 ± 1389.6				3.34 ± 0.85					
		Healthy control		39	6.77 ± 4.84	103.3 ± 41.1				2.25 ± 1.28					
37	Bayraktar [49]	β-thalassemia minor		60	26	ND			425.48 ± 73.79						
		Healthy control		60	24	ND			859.75 ± 352.86						
38	Uaprasert [50]	β-thal/HbE		68	34.1 ± 12.6	2918.7 ± 2732.9			132.4 ± 32.5				29.75 ± 15.43 ng/mL		1.55 ± 0.73
		Healthy control		38	37.2 ± 8.6	152.5 ± 128.9			178.2 ± 17.6				31.18 ± 11.32 ng/mL		1.47 ± 0.43
39	Abo-Elwafa [51]	β-thalassemia		75	11.94 ± 9.27	ND							26.28 ± 18.01%		
		Healthy control		50	12.94 ± 10.45	ND							4.78 ± 2.27%		
40	Adly [52]	β-thalassemia		40	15.4 ± 3.9	2094.5 (1340–3582)					1.97 ± 1.21	110.66 ± 47.18%			
		Healthy control		40	14.7 ± 2.1	ND					1.05 ± 0.46	71.23 ± 15.72%			
41	Chaidos [53]	β-thalassemia		10	29.9 ± 7.8	2300 (500–4964)	531± 98.12	704.75 ± 255.49						72.12 ± 27.4	3.43 ± 1.48
		Healthy control		21		ND	310.5 ± 88.35	588.75 ± 92.67						46.15 ± 11.1	2.76 ± 1.24

ND = no data, PAH = pulmonary arterial hypertension, β-TM = β-thalassemia major, β-TI = β-thalassemia intermedia, Hb-Cs = Hemoglobin Constant Spring, HbE = hemoglobin E disease, NSP = non-splenectomies, SP = splenectomies, BMT = bone marrow transplantation, and TD-β-thal *=* transfusion-dependent thalassemia.

## Supplementary Materials

The following supporting information can be downloaded at https://www.mdpi.com/article/10.3390/ijms26083842/s1.

## Abbreviations

The following abbreviations are used in this manuscript:

ICAM-1	intercellular adhesion molecule-1
VCAM-1	vascular cell adhesion molecule-1
vWF	von Willebrand factor
EMPs	endothelial microparticles
NO	nitric oxide
NOS	nitric oxide synthase
ADMA	asymmetric form of dimethylarginine
ET-1	endothelin-1
SMD	standardized mean differences
CIs	confidence intervals
