# Supplementary material for: The Relevance of Endothelial Dysfunction Biomarkers in Thalassemia Patients and Healthy Individuals: A Systematic Review and Meta-Analysis
Source: Int J Mol Sci. 2025 Apr 18;26(8):3842. doi: 10.3390/ijms26083842 (PMC12027490; doi:10.3390/ijms26083842)
Supplement: Supplementary file 1 [file ijms-26-03842-s001.zip › Supplementary Table S1. Quality assessment.pdf]

Supplementary Table S1. Quality assessment of cross-sectional studies using the AXIS tools.

	Study	Clear aims/objective	Appropriate study design	Sample size justified	Target/reference population clearly defined	Sample frame is taken from an appropriate population base	Selection process represents the target/reference population	Non-responders are addressed and categorized	Risk factors and outcomes are measured appropriately	Risk factors and outcomes are measured correctly	Clear statistical analysis	Methods sufficiently described	Basic data adequately described	Response rates raise concerns about non-response bias	Describe information about non-responder	Results internally consistent	Results are presented for all the analyses	Discussions and conclusions are justified by the results	Limitations of the study are discussed	Funding sources or conflicts of interest	Ethical approval or consent of participants attained	**Total**
1	Klaihmon [1]	Y	Y	N	Y	Y	N	N	Y	Y	Y	Y	Y	N	N	Y	Y	Y	Y	Y	Y	15
2	Banerjee [2]	Y	Y	N	Y	Y	Y	N	Y	Y	Y	Y	Y	N	N	Y	Y	Y	N	Y	Y	15
3	Helmi [3]	Y	Y	N	Y	Y	Y	N	Y	Y	Y	Y	Y	N	N	Y	Y	Y	N	Y	Y	15
4	El-shanshory [4]	Y	Y	N	Y	Y	Y	N	Y	Y	Y	Y	Y	N	N	Y	Y	Y	N	N	Y	14
5	Kyriakou [5]	N	Y	N	Y	Y	N	N	Y	Y	Y	Y	Y	N	N	Y	Y	Y	N	Y	Y	13
6	Huang [6]	Y	Y	N	Y	Y	Y	N	Y	Y	Y	Y	Y	N	N	Y	Y	Y	Y	Y	Y	16
7	Viprakasit [7	Y	Y	N	Y	Y	Y	N	Y	Y	Y	Y	Y	N	N	Y	Y	Y	N	N	Y	14
8	Suvachananonda [8]	Y	Y	N	Y	Y	N	N	Y	Y	Y	Y	Y	N	N	Y	Y	Y	N	Y	Y	14
9	Chamchoi [9]	Y	Y	N	Y	Y	N	N	Y	Y	Y	Y	Y	N	N	Y	Y	Y	Y	Y	Y	15
10	Satitthummanid [10]	Y	Y	N	Y	Y	Y	N	Y	Y	Y	Y	Y	N	N	Y	Y	Y	N	Y	Y	15
11	Manakeng [11]	Y	Y	N	Y	Y	N	N	Y	Y	Y	Y	Y	N	N	Y	Y	Y	N	Y	Y	14
12	Gursel [12]	Y	Y	N	Y	Y	Y	N	Y	Y	Y	Y	Y	N	N	Y	Y	Y	Y	Y	Y	16

13	Singer [13]	Y	Y	N	Y	Y	N	N	Y	Y	Y	Y	Y	N	N	Y	Y	Y	N	Y	Y	14
14	Pallewar [14]	Y	Y	N	Y	Y	Y	N	Y	Y	Y	Y	Y	N	N	Y	Y	Y	N	Y	Y	15
15	Aggeli [15]	Y	Y	N	Y	Y	Y	N	Y	Y	Y	Y	Y	N	N	Y	Y	Y	Y	N	Y	15
16	Al-Sabaan [16]	Y	Y	N	Y	Y	Y	N	Y	Y	Y	Y	Y	N	N	Y	Y	Y	Y	Y	Y	16
17	Chansai [17]	Y	Y	N	Y	Y	Y	N	Y	Y	Y	Y	Y	N	N	Y	Y	Y	N	Y	Y	15
18	Tantawy [18]	Y	Y	N	Y	Y	Y	N	Y	Y	Y	Y	Y	N	N	Y	Y	Y	N	Y	Y	15
19	Chamchoi [19]	Y	Y	N	Y	Y	N	N	Y	Y	Y	Y	Y	N	N	Y	Y	Y	N	Y	Y	14
20	Caprari [20]	N	Y	N	Y	Y	N	N	Y	Y	Y	Y	Y	N	Y	Y	Y	Y	N	Y	Y	14
21	El-Samee [21]	Y	Y	N	Y	Y	Y	N	Y	Y	Y	Y	Y	N	N	Y	Y	Y	N	Y	Y	15
22	Ruf [22]	Y	Y	Y	Y	Y	Y	N	Y	Y	Y	Y	Y	N	N	Y	Y	Y	N	N	Y	15
23	Sirivadhanakul [23]	Y	Y	N	Y	Y	Y	N	Y	Y	Y	Y	Y	N	N	Y	Y	Y	N	Y	Y	15
24	Atichartakarn [24]	Y	Y	N	Y	Y	Y	N	Y	Y	Y	Y	Y	N	N	Y	N	Y	N	N	Y	13
25	El-Hady [25]	Y	Y	N	Y	Y	Y	N	Y	Y	Y	Y	Y	N	N	Y	Y	Y	N	N	N	13
26	Naithani [26]	Y	Y	N	Y	Y	Y	N	Y	Y	Y	Y	Y	N	N	Y	Y	Y	N	N	Y	14
27	Srihirun [27]	Y	Y	N	Y	Y	Y	N	Y	Y	Y	Y	Y	N	N	Y	Y	Y	Y	Y	Y	16
28	Chanpeng [28]	Y	Y	N	Y	Y	Y	N	Y	Y	Y	Y	Y	N	N	Y	Y	Y	N	Y	Y	15
29	Kelaidi [29]	Y	Y	N	Y	Y	Y	N	Y	Y	Y	Y	Y	N	N	Y	Y	Y	N	N	Y	14
30	Gursel [30]	Y	Y	Y	Y	Y	Y	N	Y	Y	Y	Y	Y	N	N	Y	Y	Y	N	Y	Y	16
31	Hahalis [31]	Y	Y	Y	Y	Y	Y	N	Y	Y	Y	N	Y	N	N	Y	Y	Y	Y	Y	N	15
32	Mahdi [32]	Y	Y	N	Y	Y	Y	N	Y	Y	Y	Y	Y	N	N	Y	Y	Y	N	Y	Y	15
33	Chaliasos [33]	Y	Y	Y	Y	Y	Y	N	Y	Y	Y	Y	Y	N	N	Y	Y	Y	N	N	Y	15
34	Kanavaki [34]	Y	Y	N	Y	Y	Y	N	Y	Y	Y	Y	Y	N	N	Y	Y	Y	N	Y	N	14
35	Fayed [35]	Y	Y	N	Y	Y	Y	N	Y	Y	Y	Y	Y	N	N	Y	Y	Y	N	Y	Y	15
36	Aygüneş [36]	Y	Y	Y	Y	Y	Y	N	Y	Y	Y	Y	Y	N	N	Y	Y	Y	N	Y	Y	16

37	Bayraktar [37]	Y	Y	Y	Y	Y	Y	N	Y	Y	Y	Y	Y	N	N	Y	Y	Y	N	N	Y	15
38	Uaprasert [38]	Y	Y	N	Y	Y	Y	N	Y	Y	Y	Y	Y	N	N	Y	Y	Y	Y	Y	Y	16
39	Abo-Elwafa [39]	Y	Y	N	Y	Y	Y	N	Y	Y	Y	Y	Y	N	N	Y	Y	Y	Y	Y	Y	16
40	Adly [40]	Y	Y	Y	Y	Y	Y	N	Y	Y	Y	Y	Y	N	N	Y	Y	Y	Y	Y	Y	17
41	Chaidos [41]	N	Y	Y	Y	Y	Y	N	Y	Y	Y	Y	Y	N	N	Y	Y	Y	N	N	N	13

Y = Yes and N = No. High quality/low risk bias: $\geq$ 70% (score $\geq$ 14), Moderate quality/risk bias: 60-69% (score 12-13), Low quality/high risk bias: < 60% (score < 13).

References

1. Klaihmon P., Lertthammakiat S., Anurathapan U., Pakakasama S., Sirachainan N., Hongeng S., Pattanapanyasat K. Activated platelets and leukocyte activations in young patients with β-thalassemia/HbE following bone marrow transplantation. *Thromb Res*. **2018**, *169*, 8-14.
2. Banerjee M., Kumar A., Batra H. S., Bandyopadhyay S., Kapoor R. Are thalassemia patients oxidatively challenged? *Med J Armed Forces India*. **2019**, *75*, 383-8.
3. Helmi N., Choudhry H., Qari M., Kumosani T. A., Al-Malki A. L., Moselhy S. S., Kumosani A. T. Association of serum asymmetric dimethyl-arginine and troponin I levels as a risk of myocardial infarction in thalassemia. *Afr Health Sci*. **2018**, *18*, 720-6.
4. El-shanshory M., Badraia I., Donia A., El-Kady N., Mabrouk M. Asymmetric dimethylarginine levels in children with b-thalassemia and their correlations to tricuspid regurgitant jet velocity. *Pediatric Blood and Cancer*. **2013**, *60*, S28.
5. Kyriakou D. S., Alexandrakis M. G., Kyriakou E. S., Liapi D., Kourelis T. V., Passam F., Papadakis A. Activated peripheral blood and endothelial cells in thalassemia patients. *Ann Hematol*. **2001**, *80*, 577-83.
6. Huang Y., Long Y., Deng D., Liu Z., Liang H., Sun N., Xu Y., Lai Y., Cheng P. Alterations of anticoagulant proteins and soluble endothelial protein C receptor in thalassemia patients of Chinese origin. *Thromb Res*. **2018**, *172*, 61-6.
7. Viprakasit V., Kankirawatana S., Akarasereenont P., Durongpisitkul K., Chotewuttakorn S., Tanphaichitr V. S. Baseline levels of plasma endothelin-1 (ET-1) and changes during transfusion in thalassemic patients. *Am J Hematol*. **2002**, *70*, 260-2.
8. Suvachananonda T., Wankham A., Srihirun S., Tanratana P., Unchern S., Fucharoen S., Chuansumrit A., Sirachainan N., Sibmooh N. Decreased nitrite levels in erythrocytes of children with β-thalassemia/hemoglobin E. *Nitric Oxide*. **2013**, *33*, 1-5.
9. Chamchoi A., Srihirun S., Paiboonsukwong K., Sriwantana T., Sathavorasmith P., Pattanapanyasat K., Hirsch R. E., Schechter A. N., Sibmooh N. Decreased nitrite reductase activity of deoxyhemoglobin correlates with platelet activation in hemoglobin E/ß-thalassemia subjects. *PLoS One*. **2018**, *13*, e0203955.
10. Satitthummanid S., Uaprasert N., Songmuang S. B., Rojnuckarin P., Tosukhowong P., Sutcharitchan P., Srimahachota S. Depleted nitric oxide and prostaglandin E(2) levels are correlated with endothelial dysfunction in β-thalassemia/HbE patients. *Int J Hematol*. **2017**, *106*, 366-74.
11. Manakeng K., Prasertphol P., Phongpao K., Chuncharunee S., Tanyong D., Worawichawong S., Svasti S., Chaichompoo P. Elevated levels of platelet- and red cell-derived extracellular vesicles in transfusion-dependent β-thalassemia/HbE patients with pulmonary arterial hypertension. *Ann Hematol*. **2019**, *98*, 281-8.
12. Gursel O., Tapan S., Sertoglu E., Taşçılar E., Eker I., Ileri T., Uysal Z., Kurekci A. E. Elevated plasma asymmetric dimethylarginine levels in children with beta-thalassemia major may be an early marker for endothelial dysfunction. *Hematology*. **2018**, *23*, 304-8.
13. Singer S. T., Kuypers F., Fineman J., Gildengorin G., Larkin S., Sweeters N., Rosenfeld H., Kurio G., Higa A., Jeng M., et al. Elevated tricuspid regurgitant jet velocity in subgroups of thalassemia patients: insight into pathophysiology and the effect of splenectomy. *Ann Hematol*. **2014**, *93*, 1139-48.
14. Pallewar T. S., Sharma K., Sharma S., Chandra J., Nangia A. Endothelial Activation Markers in Polytransfused Children with Beta Thalassemia: Study from a Tertiary Care Centre in India. *Indian J Hematol Blood Transfus*. **2022**, *38*, 178-83.

15. Aggeli C., Antoniades C., Cosma C., Chrysohoou C., Tousoulis D., Ladis V., Karageorga M., Pitsavos C., Stefanadis C. Endothelial dysfunction and inflammatory process in transfusion-dependent patients with beta-thalassemia major. *Int J Cardiol*. **2005**, *105*, 80-4.
16. Al-Sabaan K., Al-Awadhi A. Evaluating von Willebrand factor and ADAMTS13 levels in thalassemia major patients and assessing a possible association with Thrombospondin-1. *Int J Lab Hematol*. **2023**, *45*, 945-52.
17. Chansai S., Fucharoen S., Fucharoen G., Jetsrisuparb A., Chumpia W. Elevations of Thrombotic Biomarkers in Hemoglobin H Disease. *Acta Haematol*. **2018**, *139*, 47-51.
18. Tantawy A., Adly A., Ismail I. Endothelial nitric oxide synthase gene introne4 VNTR polymorphism in sickle cell disease and transfusion-dependent b-Thalassemia major: Relation to cardio-vascular complications. *Haematologica*. **2013**, *98*, 174-5.
19. Chamchoi A., Srihirun S., Paiboonsukwong K., Sriwantana T., Kongkaew P., Fucharoen S., Pattanapanyasat K., Sibmooh N. Hemoglobin-bound platelets correlate with the increased platelet activity in hemoglobin E/β-thalassemia. *Int J Lab Hematol*. **2020**, *42*, 518-25.
20. Caprari P., Profumo E., Massimi S., Buttari B., Riganò R., Regine V., Gabbianelli M., Rossi S., Risoluti R., Materazzi S., et al. Hemorheological profiles and chronic inflammation markers in transfusion-dependent and non-transfusion- dependent thalassemia. *Front Mol Biosci*. **2022**, *9*, 1108896.
21. Abd El-Samee H., Bassiouny N., Nabih N. Impact of activated monocyte and endothelial dysfunction on coagulopathy in Egyptian adult beta thalassemic patients. *Hematol Rep*. **2020**, *12*, 8365.
22. Ruf A., Pick M., Deutsch V., Patscheke H., Goldfarb A., Rachmilewitz E. A., Guillin M. C., Eldor A. In-vivo platelet activation correlates with red cell anionic phospholipid exposure in patients with beta-thalassaemia major. *Br J Haematol*. **1997**, *98*, 51-6.
23. Sirivadhanakul P., Chuansumrit A., Songdej D., Kadegasem P., Wongwerawattanakoon P., Sirachainan N. Increased endothelial activation in α-thalassemia disease. *Ann Hematol*. **2019**, *98*, 1593-602.
24. Atichartakarn V., Chuncharunee S., Archararit N., Udomsubpayakul U., Aryurachai K. Intravascular hemolysis, vascular endothelial cell activation and thrombophilia in splenectomized patients with hemoglobin E/β-thalassemia disease. *Acta Haematol*. **2014**, *132*, 100-7.
25. El-Hady S. B., Farahat M. H., Atfy M., Elhady M. A. Nitric oxide metabolites and arginase I levels in β-thalassemic patients: an Egyptian study. *Ann Hematol*. **2012**, *91*, 1193-200.
26. Naithani R., Chandra J., Bhattacharjee J., Verma P., Narayan S. Peroxidative stress and antioxidant enzymes in children with beta-thalassemia major. *Pediatr Blood Cancer*. **2006**, *46*, 780-5.
27. Srihirun S., Tanjararak N., Chuncharunee S., Sritara P., Kaewvichit R., Fucharoen S., Pattanapanyasat K., Sibmooh N. Platelet hyperactivity in thalassemia patients with elevated tricuspid regurgitant velocity and the association with hemolysis. *Thromb Res*. **2015**, *135*, 121-6.
28. Chanpeng P., Svasti S., Paiboonsukwong K., Smith D. R., Leecharoenkiat K. Platelet proteome reveals specific proteins associated with platelet activation and the hypercoagulable state in β-thalassmia/HbE patients. *Sci Rep*. **2019**, *9*, 6059.
29. Kelaidi C., Kattamis A., Apostolakou F., Poziopoulos C., Lazaropoulou C., Delaporta P., Kanavaki I., Papassotiriou I. PlGF and sFlt-1 levels in patients with non-transfusion-dependent thalassemia: Correlations with markers of iron burden and endothelial dysfunction. *Eur J Haematol*. **2018**, *100*, 630-5.
30. Gursel O., Kurekci A. E., Tascilar E., Ileri T., Altun D., Tapan S., Kurt I., Kocaoglu M., Aydin A., Okutan V., et al. Premature atherosclerosis in children with β-thalassemia major. *Journal of Pediatric Hematology/Oncology*. **2012**, *34*, 630-4.
31. Hahalis G., Kalogeropoulos A., Terzis G., Tselepis A. D., Kourakli A., Mylona P., Grapsas N., Alexopoulos D. Premature atherosclerosis in non-transfusion-dependent β-thalassemia intermedia. *Cardiology*. **2011**, *118*, 159-63.
32. Mahdi Z. N., Al-Mudallal S. S., Hameed B. M. Role of red blood cells "annexin V" and platelets "P-selectin" in patients with thalassemia. *Hematol Oncol Stem Cell Ther*. **2019**, *12*, 15-8.
33. Chaliasos N., Challa A., Hatzimichael E., Koutsouka F., Bourantas D. K., Vlahos A. P., Siamopoulou A., Bourantas K. L., Makis A. Serum adipocytokine and vascular inflammation marker levels in Beta-thalassaemia major patients. *Acta Haematol*. **2010**, *124*, 191-6.

34. Kanavaki I., Makrythanasis P., Lazaropoulou C., Tsironi M., Kattamis A., Rombos I., Papassotiriou I. Soluble endothelial adhesion molecules and inflammation markers in patients with beta-thalassemia intermedia. *Blood Cells Mol Dis*. **2009**, *43*, 230-4.
35. Fayed M. A., Abdel-Hady H. E., Hafez M. M., Salama O. S., Al-Tonbary Y. A. Study of platelet activation, hypercoagulable state, and the association with pulmonary hypertension in children with β-thalassemia. *Hematol Oncol Stem Cell Ther*. **2018**, *11*, 65-74.
36. Aygüneş U., Can Ü., Doğan M. T., Keçeli M., Eker H. K. The Effect of Plasma Endocan and Asymmetric Dimethyl Arginine Levels on Endothelial and Cardiac Functions in Children with Beta-Thalassemia Major. *Guncel Pediatri*. **2022**, *20*, 78-87.
37. Bayraktar N., Erkurt M. A., Aydoğdu I., BaŞflaran Y. The levels of nitric oxide in beta-thalassemia minor. *Turkish Journal of Hematology*. **2008**, *25*, 187-9.
38. Uaprasert N., Satitthummanid S., Akkawat B., Sutcharitchan P., Rojnuckarin P. Vascular and hemostatic alterations associated with pulmonary hypertension in β-thalassemia hemoglobin E patients receiving regular transfusion and iron chelation. *Thromb Res*. **2019**, *174*, 104-12.
39. Abo-Elwafa H. A., Youseff L. M., Mahmoud R. A., Elbadry M. I., Tawfeek A., Aziz S. P. Venous Thromboembolism Risk Assessment among Beta-thalassemia Patients. *Journal of Applied Hematology*. **2023**, *14*.
40. Adly A. A. M., El-Sherif N. H., Ismail E. A. R., El-Zaher Y. A., Farouk A., El-Refaey A. M., Wahba M. S. Vascular Dysfunction in Patients With Young β-Thalassemia:Relation to Cardiovascular Complications and Subclinical Atherosclerosis. *Clinical and Applied Thrombosis/Hemostasis*. **2015**, *21*, 733-44.
41. Chaidos A., Makis A., Hatzimichael E., Tsiara S., Gouva M., Tzouvara E., Bourantas K. L. Treatment of beta-thalassemia patients with recombinant human erythropoietin: effect on transfusion requirements and soluble adhesion molecules. *Acta Haematol*. **2004**, *111*, 189-95.
